# A Serious Game for Clinical Assessment of Cognitive Status: Validation Study

**DOI:** 10.2196/games.5006

**Published:** 2016-05-27

**Authors:** Tiffany Tong, Mark Chignell, Mary C. Tierney, Jacques Lee

**Affiliations:** ^1^ Interactive Media Lab Department of Mechanical and Industrial Engineering University of Toronto Toronto, ON Canada; ^2^ Knowledge Media Design Institute Faculty of Information University of Toronto Toronto, ON Canada; ^3^ Department of Family and Community Medicine University of Toronto Toronto, ON Canada; ^4^ Primary Care Research Unit Sunnybrook Health Sciences Centre Toronto, ON Canada; ^5^ Clinical Epidemiology Unit and Department of Emergency Services Sunnybrook Health Sciences Center Toronto, ON Canada

**Keywords:** cognitive assessments, cognitive screening tools, computerized assessments, games, human computer interaction, human factors, neuropsychological tests, screening, serious games, tablet computers, technology assessment, usability, validation studies, video games

## Abstract

**Background:**

We propose the use of serious games to screen for abnormal cognitive status in situations where it may be too costly or impractical to use standard cognitive assessments (eg, emergency departments). If validated, serious games in health care could enable broader availability of efficient and engaging cognitive screening.

**Objective:**

The objective of this work is to demonstrate the feasibility of a game-based cognitive assessment delivered on tablet technology to a clinical sample and to conduct preliminary validation against standard mental status tools commonly used in elderly populations.

**Methods:**

We carried out a feasibility study in a hospital emergency department to evaluate the use of a serious game by elderly adults (N=146; age: mean 80.59, SD 6.00, range 70-94 years). We correlated game performance against a number of standard assessments, including the Mini-Mental State Examination (MMSE), Montreal Cognitive Assessment (MoCA), and the Confusion Assessment Method (CAM).

**Results:**

After a series of modifications, the game could be used by a wide range of elderly patients in the emergency department demonstrating its feasibility for use with these users. Of 146 patients, 141 (96.6%) consented to participate and played our serious game. Refusals to play the game were typically due to concerns of family members rather than unwillingness of the patient to play the game. Performance on the serious game correlated significantly with the MoCA (r=–.339, *P* <.001) and MMSE (r=–.558, *P* <.001), and correlated (point-biserial correlation) with the CAM (r=.565, *P* <.001) and with other cognitive assessments.

**Conclusions:**

This research demonstrates the feasibility of using serious games in a clinical setting. Further research is required to demonstrate the validity and reliability of game-based assessments for clinical decision making.

## Introduction

The rapidly aging population and high prevalence of age-related conditions, such as delirium and dementia, are placing increasing burdens on health care systems (eg, [1]). More frequent and accessible methods for cognitive screening are needed to detect early signs of impairment and to prevent or better manage further decline. We envision future development of independent patient-administered methods of cognitive screening that can be completed within a hospital or home. Demonstrating that serious games are highly correlated with other methods of cognitive assessment is necessary but not sufficient to justify their use. In order to ensure adequate motivation and realistic assessment of ability, game-based cognitive assessments should be interactive and engaging. They should also be enjoyable so that patients are willing to complete the assessment task at regular intervals.

### Background

In geriatric health care, there are standard mental status tools that screen for cognitive impairment, such as the Mini-Mental State Examination (MMSE) [2], Montreal Cognitive Assessment (MoCA) [3], and Confusion Assessment Method (CAM) [4].

Current cognitive screening methods are only minimally interactive, creating little in the way of engagement or entertainment. They are typically initiated by a health care professional rather than sought out by individuals and they are generally not designed for self-administration or for use by nonclinicians. Some tools such as the CAM require subjective assessments, which may result in administrator bias [4]. Additionally, it may not be feasible for the test administrator to repeatedly assess individuals for changes in their cognitive status over time. The resulting lack of frequent assessment may result in underdiagnosis of a condition such as delirium, where cognitive status can fluctuate widely over the course of a day, making it difficult to detect early stages of delirium and initiate preventive interventions [4].

Software suites, such as CogTest [5] and the Cambridge Neuropsychological Test Automated Battery [6], offer computerized versions of traditional cognitive tests. In addition to validation issues when moving a test to the computer medium, there is also the problem of potential lack of motivation when performing somewhat uninteresting tasks on a computer. To deal with the lack of motivation and engagement, games have been promoted as a way to stimulate cognitive activity in elderly users [7] and to improve brain fitness or to preserve cognitive status. For example, the Games to Train and Assess Impaired Persons game suite is composed of eight different games to evaluate motor and cognitive abilities in individuals with impairments [8]. However, such games do not yet provide validated cognitive assessment, have not been used in the health care setting, and evidence about whether they improve broader measures of intelligence is mixed (eg, [9]).

Manera et al [10] performed a pilot study with a serious game involving patients with mild cognitive impairment (MCI) and Alzheimer disease. They were able to demonstrate that their game correlates with the MMSE and other assessments such as the Trail Making Test Part 2 and Victoria Stroop Test. Because this research [10] was carried out on patients with MCI and dementia, and involved a relatively small pilot sample of 21 people using a kitchen and cooking game, there remains a need for a validated game-like screening tool that can be completed rapidly and independently (or with minimal assistance) by a broad range of older adults with varying cognitive ability.

Serious games are games designed with a primary purpose other than entertainment, such as education and training [11]. Specially adapted games can be leveraged to create an interactive and engaging tool that promotes patient-centered cognitive assessment. Mobile phones and tablets are commonly used devices and can be used as platforms for serious gaming. Previous work has demonstrated that elderly users can use mobile phones [12, 13] and touch-based tablets [14]. Many of these technologies also provide the ability to modify contrast/brightness and text size/font to increase readability. Gaming on mobile platforms is already a growing trend that is enjoyed across a wide range of age groups. Thus, the design of a game-based assessment on a mobile platform would likely increase the accessibility of cognitive assessment.

Although there are many potential benefits of designing games for the elderly, there are possible shortcomings to consider. For instance, some elderly users may not be interested in playing games or may be uncomfortable using technology [8]. A brief comparison between paper-and-pencil–based methods and serious games for cognitive assessment is provided in Table 1.

**Table 1:** Comparison between traditional paper-and-pencil cognitive assessments and the use of serious games for cognitive screening.

Feature	Paper-based assessments	Serious games
Administration method	Trained administrator	Self
Administration bias potential	Yes	No
Equipment	Paper, pencil	Tablet
Repeatability	Limited repeatability—not necessarily if alternate forms are available	Yes
Multiple variations	Few or none	Yes, can be randomized
Motivation/Entertainment	Low	High, if target users enjoy playing the game
Validation	Available	Yet to be completed

Serious games have been used in health care for the purpose of brain training in projects such as the ElderGames [7], Smart Aging [15], and the work reported by Anguera et al [16]. The ElderGames project uses a large touchscreen tabletop surface as a gaming platform. The goal of this work is to promote social interactions through gameplay with other elderly adults. A limitation associated with this work is that it requires a large apparatus and is not mobile. Moreover, the Smart Aging platform uses a computer and touchscreen monitor to simulate a virtual loft apartment. It is designed to identify MCI through the completion of a series of tasks that simulate daily activities [15]. This project was reported to be in the pilot phase and was evaluated with a relatively small sample of healthy individuals (N=50). A computer-based serious game has been created [16] that simulates driving a vehicle. However, that research compared serious game performance in elderly users with their performance on psychological tasks rather than with standard cognitive assessments. In contrast, we are explicitly developing a game for cognitive assessment.

### Development of a Serious Game

**Figure 1:**
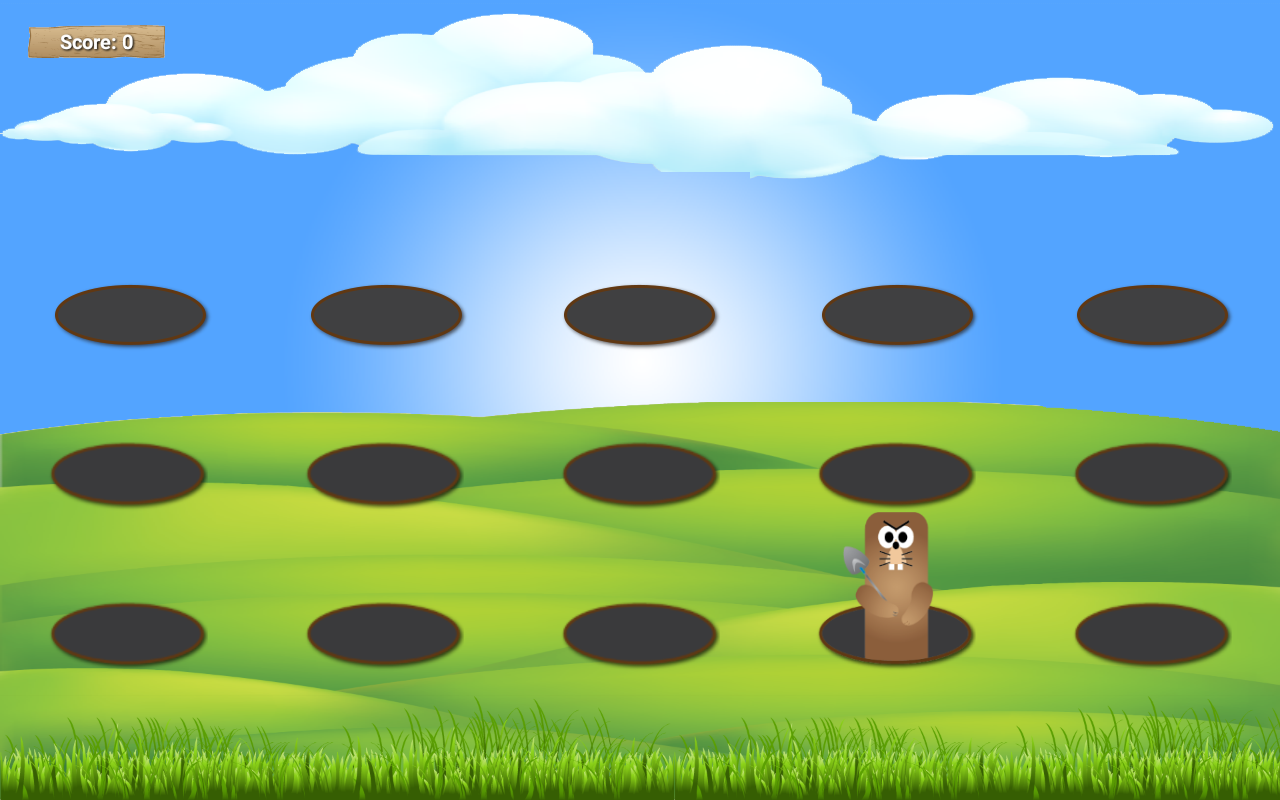
Screenshot of the whack-a-mole game.

We developed a serious game to assess cognitive status in elderly adults with a focus on detecting small changes in cognition for conditions such as delirium. Our serious game mimics features of the classic psychological Go/No-Go Discrimination Task [17], a measure of inhibition ability. As implemented, our game is similar to the carnival game whack-a-mole (see Figure 1). In a previous study with healthy younger adults, we found that our serious game had a significant relationship (*r* =.60, *P* <.001) with the Stroop task [14]. The Stroop task is a test of the inhibitory executive function, which declines with age, and the task has been shown to correlate with white matter loss in the brain [18, 19].

After demonstrating that the game-based screening tool was usable by young and older healthy adult samples, and was predictive of inhibition ability, our next step was to evaluate its usability in a clinical sample. In this paper, we present our findings concerning the process of integrating a game-based cognitive assessment into a clinical environment. We demonstrate that our serious game is usable by an elderly population from an emergency department (ED) and is predictive of scores on standard cognitive assessments. The ED is a promising target for serious game-based cognitive assessment because there is a high prevalence of cognitive impairment in that setting compounded by a high rate of underdetection of delirium [20]. Based on the findings from this research, a set of design guidelines is provided in a later section of this paper to assist future researchers in implementing other serious games for assessing cognitive ability.

## Methods

**Figure 2:**
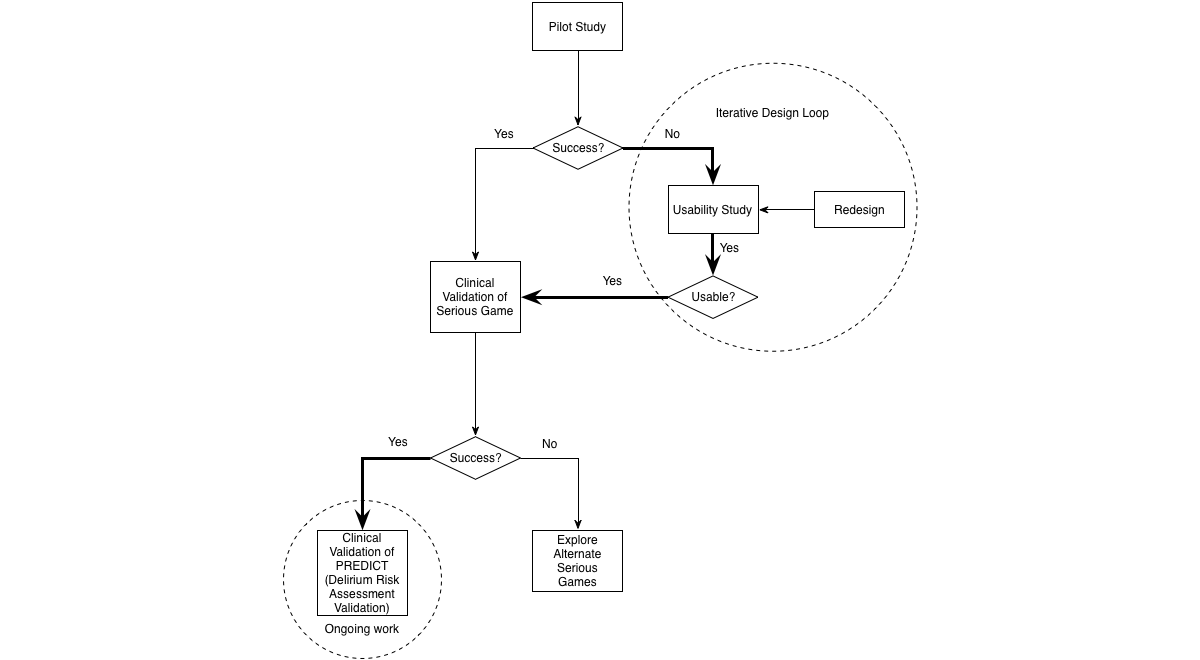
Diagram of studies in this research. The thick line highlights the path taken in this study.

We conducted a prospective observational clinical study with participants recruited from the Sunnybrook Health Sciences Centre ED (see Figure 2) located in Toronto, Ontario, Canada under a research protocol approved by both the Research Ethics Boards of the Sunnybrook Health Sciences Centre and the University of Toronto. Participants who were 70 years or older and who were present in the ED for a minimum of 4 hours were recruited for the study. Exclusion criteria included patients who were (1) critically ill (defined by the Canadian Triage Acuity Scale score of 1), (2) in acute pain (measured using the Numeric Rating Scale with a score greater than or equal to two out of 10), (3) receiving psychoactive medications, (4) judged to have a psychiatric primary presenting complaint, (5) previously enrolled, (6) blind, or (7) unable to speak English, follow commands, or communicate verbally.

Clinical research assistants (RAs) administered standard cognitive assessments including the MMSE, CAM, Delirium Index (DI) [21], Richmond Agitation-Sedation Scale (RASS) [22], Digit Vigilance Test (DVT) [23], and a choice reaction time (CRT) task. Each participant was then asked to play the serious game and provide feedback. The serious game was played on a 10-inch Samsung Galaxy Tab 4 10.1 tablet. Participants received instructions on how to play the game and interact with the tablet. There was no limit on the number of attempts to play the game. Participants were invited to provide open feedback at the end of the study. At the end of each session, the RA informally interviewed the participant on his/her experience with the game. In addition, RAs provided their own feedback and comments on their experience with the game and their observations of the interaction between each participant and the game.

The RAs recorded the date of the ED visit, whether the cognitive assessments were refused, and the cognitive assessment scores. Usage notes were also recorded and later used to infer usability problems as well as evidence for enjoyment and engagement.

### Statistical Analysis

The cognitive data and serious game results were nonnormally distributed based on visual inspection of the data. Tests for normality, including the Kolmogorov-Smirnov and Shapiro-Wilk tests [24], were not used due to the large sample size in this study because they are known to result in oversensitivity to relatively small departures from normality [24]. Transformations of the data were not performed because some of the measures, such as the CAM and DI, are binary/categorical and cannot follow a normal distribution. Our interest was in correlations as a measure of the effect size of the underlying relationship between game performance and the cognitive assessments, but we used nonparametric correlation measures for some of the comparisons [25] that involved categorical or narrow ordinal scales. Correlations between the dichotomous CAM and the other measures were assessed using point-biserial correlations [24]. Correlations involving the DI and RASS (and not involving the CAM) were assessed using Spearman rho because the DI and RASS use a small number of ordered categories. The remaining comparisons were done using Pearson correlations. In order for readers to judge strengths of relationships involving game performance, scatterplots of the relationship between game performance and the MMSE, MoCA, and CAM, respectively, are also presented.

## Results

### Description of Sample

We recruited 147 participants (80 males and 67 females) between the ages of 70 and 94 years (mean 80.61, SD 6.08). One participant was excluded for not completing any of the cognitive assessments and five people did not play the serious game (of whom two were CAM-positive), leaving 141 participants who completed the study (age range 70-94, mean age 80.64, SD 6.09; 79 males and 67 females).

Some participants declined to complete some of the cognitive assessments entirely or declined to answer certain questions. The completion rate of each test is shown in Table 2. All participants completed the CAM, DI, and RASS. The serious game had a combined completion rate of 96.6% (141/146), whereas the completion rates for the other assessments were lower with DVT being the worst at 36.3% (37/102) overall. Because the DVT and CRT assessments were initiated partway through the study, the denominators in calculating completion rates for those measures (102 and 99, respectively) were lower than for the other tests (which were initiated at the start of the study).

**Table 2:** Summary of completion rates for standard cognitive assessment scores.

Cognitive assessment	Completion rate, n (%)
Mini-Mental State Examination (MMSE)	145/146 (99.3)
Montreal Cognitive Assessment (MoCA)	108/146 (73.9)
Confusion Assessment Method (CAM)	146/146 (100.0)
Delirium Index (DI)	146/146 (100.0)
Richmond Agitation-Sedation Scale (RASS)	146/146 (100.0)
Digit Vigilance Test (DVT)^a^	37/102 (36.3)
Choice Reaction Task (CRT)^a^	82/99 (83)
Serious game	141/146 (96.6)

^a^This assessment was introduced later in the study.

There were a number of people in the sample with low MMSE and MoCA scores (down to 9 and 8, respectively). There were 129 participants who were negative for the CAM and 12 participants who were positive (a positive result on the CAM suggests that the participant has delirium). Moreover, the DI scores ranged from 0 to 10 (the score indicates the severity of delirium), RASS scores ranged from –2 to 1 (a score >0 suggests that the patient is agitated and a score <0 suggests that the patient is sedated), DVT scores ranged from 81 to 103, and CRT choice accuracy ranged from 34% to 95%. The combined median response time (RT) on the CRT was 1.2 sec (IQR 0.4). The overall median RT on the serious game was 0.9 sec (IQR 0.2), and the mean accuracy was a deviation of 328.5 pixels (SD 59.7) from the center of the target. A summary of the scores on the cognitive assessments can be found in Table 3.

**Table 3:** Summary of study sample demographics and cognitive assessment scores.

Variable	Males (n=80)		Females (n=66)		Total (N=146)	
	Mean (SD) / median (IQR)^a^	Range	Mean (SD) / median (IQR)^a^	Range	Mean (SD) / median (IQR)^a^	Range
Age (years)	80.6 (6.3)	70-94	80.6 (5.7)	70-94	80.6 (6.0)	70-94
MMSE	28.2 (1.5)	25-30	27.7 (2.2)	9-30	26.7 (3.9)	9-30
MoCA	24.5 (2.6)	8-30	23.2 (3.8)	15-30	23.2 (4.6)	8-30
CAM	0.1 (0.3)	0-1	0.1 (0.3)	0-1	0.1 (0.3)	0-1
DI	0.5 (0.7)	0-10	0.5 (0.8)	0-8	1.3 (2.3)	0-10
RASS	–0.1 (0.4)	–2 to 1	–0.1 (0.4)	–2 to 1	–0.1 (0.3)	–2 to 1
DVT	97.5 (5.7)	81-103	98.7 (4.0)	92-103	97.8 (5.3)	81-103
CRT RT (sec)	1.2 (0.3)	0.87-1.98	1.2 (0.5)	0.78-3.23	1.2 (0.4)	0.78-3.40
CRT accuracy (%)	87 (1)	50-95	87 (13)	34-95	87 (1)	34-95
Game RT (sec)	0.8 (0.1)	0.65-2.46	0.9 (0.3)	0.65-2.65	0.9 (0.2)	0.65-2.65
Game accuracy (pixels)	331.9 (49.0)	140-449	327.8 (69.9)	81-424	328.5 (59.7)	81-449

^a^For CRT RT and game RT, the median (IQR) is reported. All others are mean (SD).

### Comparison Between Serious Game Performance and Standard Cognitive Assessments

Game performance was measured based on a participant’s RT and accuracy. In our serious game, RT was measured from the time the target appeared to the time of the user’s response and accuracy was measured as the pixel distance between the center of the target and the center of the user’s touch.

Correlation analysis revealed significant relationships between game median RT and scores on the six cognitive assessments: MMSE, MoCA, CAM, DI, RASS, DVT, and CRT RT (see Table 4). In contrast to the RT results, the corresponding relationships between game accuracy and the standard cognitive assessments were not statistically significant, except for the relationship with DVT. Note that information about which types of correlation were used for each comparison are shown in the footnotes to Table 4.

**Table 4:** Correlations comparing game performance to the standard cognitive assessments..

Measure	Correlation^a^(*P*-value)									
	Game RT	Game accuracy	MMSE	MoCA	CAM	DI	RASS	DVT	CRT RT	CRT accuracy
Game RT	1	.132 (.12)	–.558 (<.001)	–.339 (<.001)	.565 (<.001)	.280 (<.001)	–.296 (<.001)	–.122 (.48)	.625 (<.001)	–.325 (.003)
Game accuracy		1	–.104 (.22)	–.042 (.67)	.071 (.40)	.048 (.46)	–.108 (.12)	.432 (.008)	–.053 (.64)	.004 (.97)
MMSE			1	.630 (<.001)	–.693 (<.001)	–.689 (<.001)	.339 (<.001)	.200 (.24)	–.503 (<.001)	.307 (.005)
MoCA				1	–.505 (<.001)	–.339 (<.001)	.193 (.01)	.192 (.28)	–.296 (.01)	.148 (.22)
CAM					1	.515 (<.001)	–.644 (<.001)	—^b^	.434 (<.001)	–.237 (.03)
DI						1	–.418 (<.001)	–.037 (.79)	.272 (.002)	–.160 (.06)
RASS							1	—^b^	–.124 (.17)	.129 (.16)
DVT								1	.045 (.80)	–.237 (.18)
CRT RT									1	–.503 (<.001)
CRT accuracy										1

^a^Correlations involving the CAM were calculated using point-biserial correlations. Correlations involving the DI and RASS (and not involving the CAM) were assessed using Spearman rho. All other correlations were calculated using Pearson *r*.

^b^Cannot be computed because at least one of the variables is constant.

As a follow-up to our correlation analyses in Table 4, we carried out the same analysis using Spearman rho correlations instead of Pearson correlations. All significant correlations between the cognitive assessments and game RT and game accuracy, respectively, were also observed to be significant using Spearman rho.

In order to examine the separate contributions of speed of processing and executive functioning on cognitive assessment scores, we looked at the partial correlations of serious game and CRT performance (controlling for each other) with the clinical assessments (see Table 5). The partial correlations with game RT (controlling for CRT) remained significant for the MMSE, CAM, and DI, but not for the MoCA and DVT. There was one significant relationship for the partial correlation of game accuracy (controlling for CRT) with DVT. On the other hand, the partial correlations involving CRT, but controlling for serious game performance RT, were not significant except for the MMSE (see Table 5). In addition, the partial correlations involving CRT but controlling for game accuracy were significant for the DI only (Table 5).

**Table 5:** Partial correlations that control for CRT RT on game performance and standard cognitive assessments and control for game RT on standard cognitive assessments.

Assessment	Control for CRT RT				Control for game RT			
	Serious game median RT		Serious game median accuracy		CRT RT		CRT Accuracy	
	ρ	*P*	ρ	*P*	ρ	*P*	ρ	*P*
MMSE	–.313	.005	–.024	.84	–.241	.03	.221	.52
MoCA	–.068	.58	.160	.19	–.197	.11	.063	.61
CAM	.516	<.001	–.112	.33	–.040	.73	.014	.01
DI	.412	<.001	.066	.56	.215	.06	–.255	.02
RASS	.173	.13	–.088	.44	–.179	.11	.135	.24
DVT	.39	.440	.01	.105	.57	–.227	.21	–.159

### Detection of Abnormal State Using Serious Game Performance

**Figure 3:**
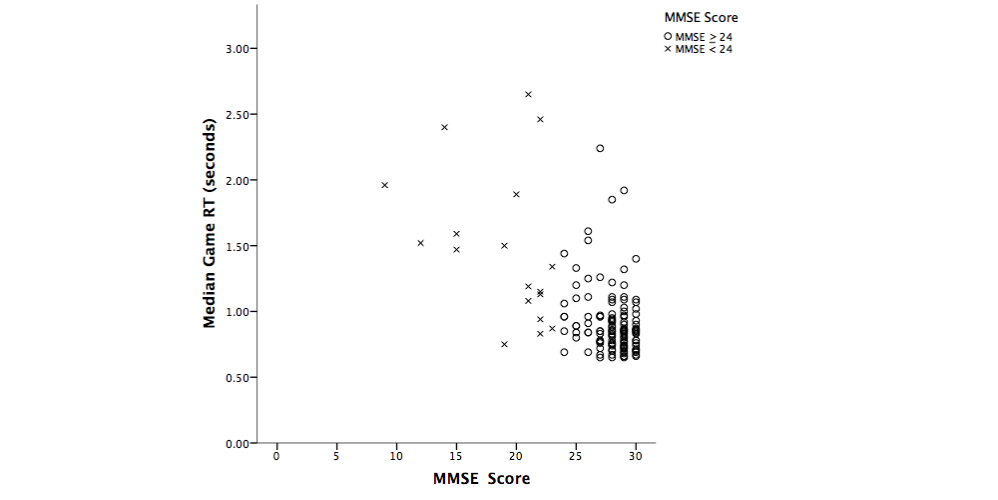
Scatterplot illustrating the differences on game RT performance based on MMSE score (≥24=normal cognitive function or possible MCI; <24=signs of dementia).

A Mann-Whitney *U* test (see Table 6) was performed to investigate the difference between cognitive ability and serious game performance when the MMSE score was 24 and above (normal cognitive function or possible MCI) versus when that score was below 24 (signs of dementia) [2, 26]. The MMSE was chosen as the grouping criterion because it was a standard in screening for dementia at the time this research was carried out. The test results suggest that there was a significant difference on the CRT in terms of RT between participants with dementia (MMSE <24) and no dementia (MMSE ≥24) [26]. In addition, there was a significant difference between MMSE groups in terms of game RT (*U* =348.5, *z* =–4.7; *P* <.001), but not for game accuracy. For Table 6, the corresponding scatterplot (Figure 3) is also shown. Figure 3 shows the distribution of game RT versus MMSE (“dementia” scores are indicated by triangles) where a tendency for lower MMSE scores to be associated with longer RTs can be seen.

**Table 6:** Mann-Whitney *U* test results comparing cognitive assessment performance based on the absence (≥24) or presence (≤24) of dementia as assessed by the MMSE.^a^

Assessment^b^	MMSE <24		MMSE ≥24		*U*	*P*	*z*	*r*	IQR
	n	Mean (SE)	n	Mean (SE)					
Game RT	18	327.6 (17.6)	122	317.2 (5.2)	348.5	<.001	–4.7	.4	0.9-1.1
CRT RT	8	2.2 (0.3)	73	1.3 (0.0)	104.0	.003	–2.9	.3	1.0-1.4
CRT accuracy	8	0.7 (0.0)	73	0.8 (0.0)	181.0	.08	–1.7	.1	0.8-0.9
Game accuracy	18	0.7 (0.0)	122	0.8 (0.0)	980.5	.46	–0.7	.0	299.0-328.5

^a^Table has been reordered based on the *U* statistic value according to estimated *P* value.

^b^RT measures are reported in seconds, CRT accuracy reflects proportion of responses that were correct, and game accuracy reflects deviation in pixels from the center of the target.

**Figure 4:**
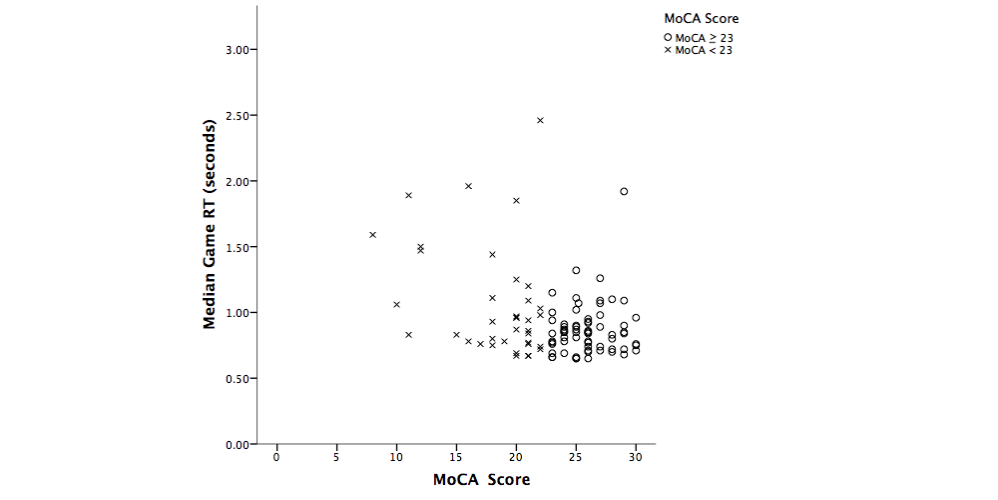
Scatterplot illustrating the differences on game RT based on MoCA score (≥23=normal cognitive function; <23=cognitive impairment).

Similar to the analysis reported in Table 6, a Mann-Whitney *U* test (see Table 7) was performed to investigate the difference between cognitive ability and serious game performance when the MoCA score was 23 and above (normal cognitive function) versus below 23 (MCI) [27]. The MoCA was chosen as the criterion in this comparison because it is a de facto standard in screening for MCI versus normality. There was a significant difference (*U* =947.5, *z* =–2.7; *P* =.001) on the CRT RT between participants with cognitive impairment (MoCA <23) and no impairment (MoCA ≥23). There was also a significant difference between MoCA groups for game RT (*U* =370.0, *z* =–3.2; *P* =.03). For Table 7, the bivariate relationship is illustrated in the scatterplot in Figure 4. This figure illustrates a tendency for lower MoCA scores to be associated with longer RTs, although that relationship appeared to be weaker for the MoCA than it was for the MMSE.

**Table 7:** Mann-Whitney *U* test results comparing game performance based on the absence (≥23) or presence (≤23) of cognitive impairment as assessed by the MoCA.^a^

Assessment^b^	MoCA, <23		MoCA ≥23		*U*	*P*	*z*	*r*	IQR
	n	Mean (SE)	n	Mean (SE)					
Game RT	38	1.0 (0.07)	67	0.9 (0.02)	307.0	.03	–3.2	.31	0.7-117
CRT RT	26	1.6 (0.1)	44	1.2 (0.08)	947.5	.001	–2.7	.32	1.0-1.1
CRT accuracy	26	0.8 (0.02)	44	0.9 (0.02)	439.5	.11	–1.6	.19	0.8-0.9
Game accuracy	38	317.5 (9.2)	67	3222.4 (5.6)	1240.0	.83	–0.2	.02	299.0-352.5

^a^Table has been reordered based on the *U* statistic value according to significance.

^b^RT measures are reported in seconds, CRT accuracy reflects proportion of responses that were correct, and game accuracy reflects deviation in pixels from the center of the target.

**Figure 5:**
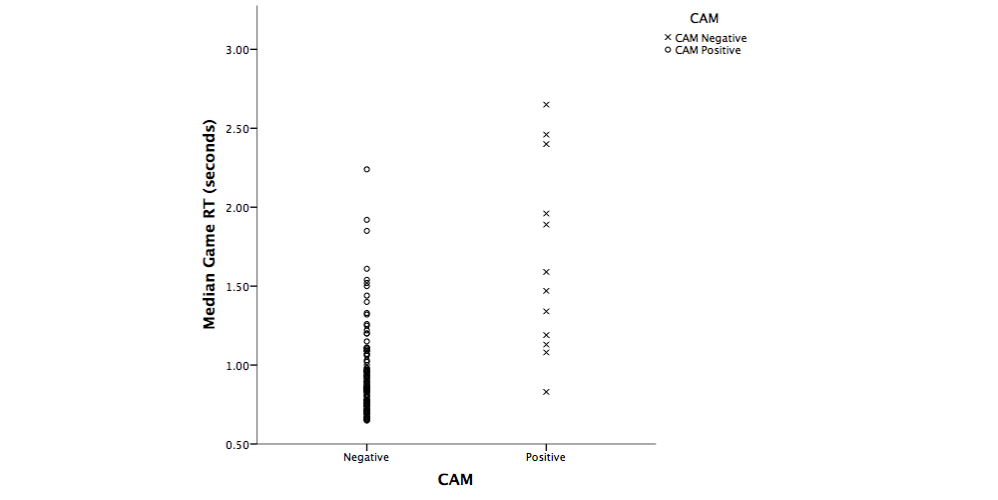
Scatterplot illustrating the differences on game RT based on CAM groups (CAM negative=delirium absent; CAM positive=delirium present).

Another Mann-Whitney *U* test (see Table 8) was performed to investigate the difference between cognitive ability and serious game performance when delirium was present (CAM positive) versus absent (CAM negative). The CAM was chosen as the grouping factor as it is the gold standard in screening for delirium. The test indicated a significant difference on the MMSE, MoCA, RASS, and DI between participants with delirium (CAM positive) and no delirium (CAM negative). In addition, there was a significant difference between CAM groups in terms of RT on the serious game (*U* =–4.5, *P* <.001). For Table 8, this relationship is shown in Figure 5. These between-group differences in game RT and MMSE are consistent with findings by Lowery [28], where CAM-negative participants demonstrated faster RT and higher MMSE scores compared to CAM-positive participants.

**Table 8:** Mann-Whitney *U* test results comparing cognitive assessment performance based on the absence (CAM negative) or presence (CAM positive) of delirium as assessed by the CAM.^a^

Assessment^b^	CAM Negative		CAM Positive		*U*	*P*	*z*	*r*	IQR
	n	Mean (SE)	n	Mean (SE)					
RASS	14	–0.03 (0.02)	142	–0.8 (0.2)	288.0	<.001	–7.8	.62	0.0-0.0
Game RT	12	0.9 (0.02)	129	1.7 (0.2)	158.0	<.001	–4.5	.38	0.7-1.1
MoCA	7	23.8 (0.4)	101	14.3 (2.0)	60.5	<.001	–3.7	.36	21.0-26.0
MMSE	14	27.6 (0.2)	131	18.4 (1.3)	38.0	<.001	–5.9	.49	26.0-29.0
DI	14	0.6 (0.1)	131	6.9 (0.5)	24.5	<.001	–6.6	.55	0.0-1.0
CRT RT	4	1.3 (0.06)	78	2.6 (0.5)	45.0	.02	–2.4	.26	1.0-1.4
CRT accuracy	4	0.8 (0.01)	78	0.7 (0.1)	91.5^c^	.17	–1.4	.15	0.8-0.9
Game accuracy	12	317.3 (5.3)	129	332.4 (15.2)	708.0	.63	–0.5	.04	299.0-352.5

^a^Table has been reordered based on the *U* statistic value according to significance. No Mann-Whitney *U* test analysis was carried out for the DVT because there were no CAM-positive participants who completed the DVT. Additional assessments are included in this table for the purpose of comparison.

^b^RT measures are reported in seconds, CRT accuracy reflects proportion of responses that were correct, and game accuracy reflects deviation in pixels from the center of the target. Other measures shown reflect the scores on the instruments (MoCA, MMSE, DI, RASS).

^c^The independent samples *t* test was nonsignificant for this comparison (*t*_80_=1.5, *P* =.21).

As a check, we replicated all the Mann-Whitney *U* tests in Tables 6-8 with their parametric equivalent, in this case the independent samples *t*-test. The pattern of significant and nonsignificant effects was identical for both tests, with the exception of the comparison of CRT RT between CAM-positive and CAM-negative participants (Table 8). For that comparison, the independent samples *t*-tests did not show a significant effect, whereas the Mann-Whitney *U* test did.

### Predicting Delirium Status Using Serious Game Performance

In the preceding section, we examined the relationship between game performance and current standards for clinical assessment with respect to MCI, delirium, and dementia. In this section, we examine the question of how well the serious game performance predicted CAM status (delirium).

Discriminant analysis was carried out to see how well game performance could predict CAM status. The two predictors were game RT and accuracy. Game accuracy provided no benefit in prediction and received a zero weight in the discriminant function. Thus, we focused on game RT as a potential screener for further evaluation using the CAM. We examined different possible cutoff values for distinguishing between people who should be screened for possible delirium (using the CAM) and those who should not.

Setting a relatively long median RT for the decision threshold (≥1.88 seconds) resulted in good specificity (127/129, 98.4% CAM-negative patients were correctly identified), but relatively poor sensitivity (only 5/12, 41% CAM-positive patients were correctly identified).

On the other hand, using a more stringent median RT cutoff of 1.13 seconds, there was both good sensitivity (10/12, 83% CAM-positive patients were correctly identified) and good specificity (114/129, 88.3% CAM-negative patients were correctly identified).

**Figure 6:**
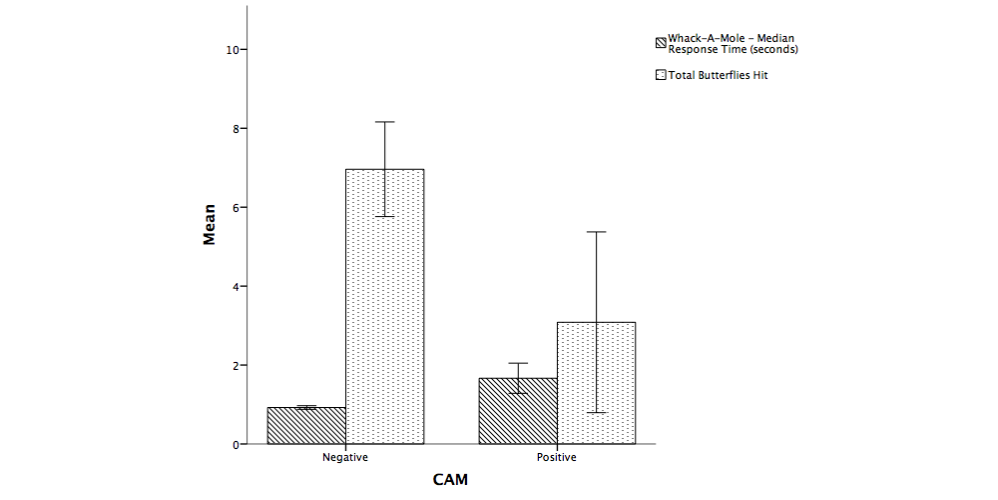
Mean of median RTs and mean number of butterflies hit for CAM-negative and CAM-positive patients. Error bars indicate 95% CI.

We also found that CAM-positive patients hit fewer distractors by mistake (as shown in Figure 6). Since CAM-positive participants had fewer hits in general (to both moles and butterflies), it seems likely that their apparently lower error rate was due to a lower response rate rather than to the presence of a speed-accuracy tradeoff.

### Usability Issues and Evidence of Enjoyment and Engagement

The following brief notes recorded by the RAs during patient use of the serious game are indicative examples of enjoyment and engagement that were observed: “Loved the game, she was playing games on her iPhone before I approached her” “Enjoyed the game, he would play on his own,” “Too easy but don’t make it too challenging, like the game,” and “Really loved the tablet, wanted to keep playing even after testing was over.” However, usability problems were also observed. Some participants placed their palm on the tablet while trying to interact with the serious game. This confused the software because it was unclear which hit points were intentional versus accidental. Some participants claimed that the game was too easy and suggested that we include more difficult levels to make it more interesting. Elderly users also expressed an interest in playing games such as crossword puzzles. Anecdotally, the RAs who supervised the data collection at the hospital reported that this game was easier to administer and more fun to complete compared to standard cognitive assessments such as the MoCA and DVT.

### Ergonomic Issues

While interacting with the tablets, the elderly participants assumed numerous positions, such as being seated, lying down, standing, or walking around. Each of these positions had different ergonomic requirements and some brief recommendations based on our experience in this study are provided in the Discussion. Some participants were also frail and required the assistance of the RA to hold the tablet for them.

## Discussion

Performance on the serious game in terms of median RT was significantly correlated with MMSE, MoCA, CAM, DI, RASS, DVT, and CRT scores for elderly ED patients and differences were in the expected direction (slower game RT for people with possible MCI and dementia). The correlations suggest a relationship between longer RT on the game and lower cognitive assessment scores. These correlations demonstrate the potential value of serious games in clinical assessment of cognitive status. The correlations between the standard cognitive tests observed in this study are similar to results seen in other research. For example, correlations of *r* =.43 and *r* =.60 between MMSE and MoCA scores for healthy controls and patients with MCI, respectively, have been found [29]. In our study, we observed a correlation of *r* =.63 (*P* <.001) between the MMSE and MoCA scores. Overall, the correlation of our serious game with existing methods of clinical cognitive assessment appears to be almost as strong as the correlations of the clinical assessment methods with themselves.

In our partial correlation analysis, we observed that our serious game correlates with the MMSE and DI, but that part of that correlation is attributable to speed of processing (CRT speed). Thus, serious game performance in this case involved both speed of processing and executive functioning components. Both components are involved in the correlation of the serious game with the MMSE. However, only the speed of processing component appears to be involved in the correlation with the MoCA. Crucially, the partial correlations of serious game performance (controlling for CRT RT) were higher than the corresponding partial correlations for CRT (controlling for serious game performance) indicating that the serious game is an overall better predictor of cognitive status than simple processing speed as measured by the CRT task.

We found that there was a lack of association between serious game accuracy and scores on cognitive assessments. This may be due to variations in interaction methods where some users used their fingers instead of a stylus to interact with the tablet device. Another reason may be that some users preferred responding more quickly over being accurate in their responses.

One of the goals of this research was to develop a method for predicting the presence of delirium using this serious game. In this study, we found that a median RT cutoff of 1.13 seconds implied relatively good sensitivity and specificity in the clinical decision. However, 25 of the 129 (19.4%) participants were above the median cutoff and only 10 of these were CAM-positive. Thus, in a clinical setting the question remains of how to deal with people who are identified as CAM-positive using this RT cutoff value. One approach would be to give those people full CAM assessment and then treat the CAM-positive patients accordingly. The value of the serious game in this case is that it would allow (based on screening with the serious game) a high rate of delirium detection using CAM assessment in only around 20% of patients (assuming that the current results generalize to other contexts). Ideally, a suitably adapted serious game would also detect risk of delirium onset so that prevention strategies could be used on targeted patients before they developed delirium, but that prospect was beyond the scope of the research reported in this paper.

During our studies, we observed many ergonomic issues that could arise during the administration of the serious game. For instance, there were a variety of positions and methods used to interact with the tablet-based serious game. For participants who are sitting down, we recommend a tablet case that has a hand holder or kickstand to allow them to interact with the tablet in multiple ways. In contrast, for participants lying down on a bed, it may be difficult for them to hold the tablet to play the serious game; thus, a stand affixed to a table or intravenous pole that holds up the tablet would be appropriate. Furthermore, the ergonomic solutions that are adopted should meet hospital standards on hygiene and sanitization for technology. For patients with hand injuries or visual disabilities, the serious game may not be a usable option.

User-centered design and ergonomic interventions were both key in ensuring that the serious game was usable with a challenging user group (elderly patients) and in the fairly unique and demanding context of a hospital ED. The touch interface was modified so that it was more forgiving of the kinds of gestures made by elderly users when interacting with the game and the gameplay was modified so that users with a wide range of ability could play the game. Ergonomic issues that were dealt with in our research included the form factor of the device and the selection and use of accessories to facilitate interactions with the device in different postures and contexts.

Based on our research experience, we present the following recommendations for enhancing tablet-based user interaction between elderly adults and touch-based technologies:

1. Accept multiple gestures, including taps and swipes, as input to maximize interaction.

2. Provide a stylus for users who have difficulties interacting with the tablet with their fingers.

3. For time-sensitive tasks, the time limit should be increased to allow older or more frail users a chance to interact with the software.

4. Tablet screen protectors should be installed to provide more friction between a user’s hand and the screen.

5. A variety of ergonomic stands and mounts should be available to accommodate various interaction positions.

6. Serious games for cognitive assessment should incorporate validated psychological task components (eg, executive functions) and should be easily playable for independent use.

7. Assess the validity of the game across different subgroups of patients. Consider the possibility of using multiple versions of a game, or multiple games, to accommodate the different characteristics and needs of different types of patient.

### Limitations

The usability and validation results obtained apply to elderly adults in an emergency setting. Further research would be needed to generalize these results to different types of patient and different clinical settings. The design of this study was cross-sectional, so each participant/patient was only studied during one ED visit and played the game only once. Future research may assess the reliability of the game when played repeatedly by the same patient in the ED. One other limitation is that only one game was examined in this research (the whack-a-mole game that we developed). Other serious games should also be explored to determine which games work best with different types of patients.

This work is an initial validation study of our serious game for cognitive screening, where the game was only administered once. One of the goals of this research is frequent cognitive screening, which can potentially lead to learning effects on the game. Future research that assesses the reliability of the game-based screening tool will need to address how to overcome and differentiate between learning effects on a patient’s game performance on our serious game versus their actual cognitive status. Because we are interested in changes in cognitive status, we are not as concerned with a patient’s improved performance due to learning effects from repeated gameplay, but would aim to track deviations in their performance over time due to cognitive decline.

### Conclusions

We believe that serious games are a promising methodology for cognitive screening in clinical settings, including the high-acuity time-pressured ED environment. This work demonstrates the feasibility of implementing a serious game for cognitive screening in a health care environment. To the best of our knowledge, this is the first time that a serious game for cognitive assessment has been tested in an ED and with a full battery of standard cognitive assessment methods for comparison. Based on these results, ergonomically appropriate serious games can potentially revolutionize cognitive assessment of the elderly in clinical settings, allowing assessments to be more frequent, more affordable, and more enjoyable.

This research provides a case study in the development of an interactive serious game for cognitive screening that may be used independently and repeatedly, thus promoting patient-centered health and safety. We have demonstrated in this study that elderly adults older than age 70 years can successfully play our serious game in an ED and that RT performance on the game can be used as an initial screen for cognitive status.

These findings do not yet demonstrate that the serious game evaluated here is ready to be used to screen for delirium in the ED. Only 12 CAM-positive patients were observed in the study and of the game performance measures (RT, accuracy, number of targets hit, number of distractors hit), only game RT was predictive of CAM status. However, due to the known underreporting of delirium in the ED, an efficient and usable method of screening for delirium is clearly needed. In this study, a game median RT cutoff of 1.13 seconds produced a sensitivity of 83% and a specificity of 88% when used retrospectively as a screen for CAM-positive status. Although further research is needed, it seems possible that a suitably revised and validated game might be able to identify approximately 80% to 90% of CAM-positive cases while requiring the screening of no more than approximately 20% of cases.

Outside the ED, the use of the serious game for ongoing patient-administered assessment would ideally involve patients who remain actively engaged with their support network (eg, family and care providers) and with health care professionals. For instance, if patients perform poorly on the serious game or notice a decline in their performance, they could discuss these results with their care providers, which might lead to interventions such as changes to medication or lifestyle that could slow observed declines.

## Abbreviations

CAM: Confusion Assessment Method
CRT: choice reaction time
DI: Delirium Index
DVT: Digit Vigilance Test
ED: emergency department
MCI: mild cognitive impairment
MMSE: Mini-Mental State Examination
MoCA: Montreal Cognitive Assessment
RA: research assistant
RASS: Richmond Agitation-Sedation Scale
RT: response time
